# Reproducibility and Reliability of the Quality of Life Questionnaire in Patients With Atrial Fibrillation

**DOI:** 10.5935/abc.20160026

**Published:** 2016-03

**Authors:** Rita Simone Lopes Moreira, Lucas Bassolli, Enia Coutinho, Paloma Ferrer, Érika Olivier Bragança, Antonio Carlos Camargo Carvalho, Angelo Amato de Paola, Bráulio Luna Filho

**Affiliations:** Universidade Federal de São Paulo, São Paulo, SP - Brazil

**Keywords:** Atrial Fibrillation/psychology, Quality of Life, Questionnaires

## Abstract

**Background:**

Studies have shown the impact of atrial fibrillation (AF) on the patients' quality of life. Specific questionnaires enable the evaluation of relevant events. We previously developed a questionnaire to assess the quality of life of patients with AF (AFQLQ version 1), which was reviewed in this study, and new domains were added.

**Objective:**

To demonstrate the reproducibility of the AFQLQ version 2 (AFQLQ v.2), which included the domains of fatigue, illness perception and well-being.

**Methods:**

We applied 160 questionnaires (AFQLQ v.2 and SF-36) to 40 patients, at baseline and 15 days after, to measure inter- and intraobserver reproducibility. The analysis of quality of life stability was determined by test-retest, applying the Bartko intraclass correlation coefficient (ICC). Internal consistency was assessed by Cronbach's alpha test.

**Results:**

The total score of the test-retest (n = 40) had an ICC of 0.98 in the AFQLQ v.2, and of 0.94 in the SF36. In assessing the intra- and interobserver reproducibility of the AFQLQ v.2, the ICC reliability was 0.98 and 0.97, respectively. The internal consistency had a Cronbach's alpha coefficient of 0.82, compatible with good agreement of the AFQLQ v.2.

**Conclusion:**

The AFQLQ v.2 performed better than its previous version. Similarly, the domains added contributed to make it more comprehensive and robust to assess the quality of life of patients with AF.

## Introduction

Atrial fibrillation (AF) is the most common sustained arrhythmia, being associated with a substantial increase in morbidity and mortality. Its prevalence increases with age, and 70% of the population with AF belongs to the age group between 65 and 85 years. It manifests more commonly in men, but after the age of 75 years, women constitute 70% of the population affected.^1,2^

In Brazil, 1.5 million individuals are estimated to have AF, which accounts for 33% of the hospitalizations. In the United States, persistent and paroxysmal AF is present in approximately 2-3 million North-Americans. In Europe, it is detected in 4.5 million people, with a projection that by 2050 it reaches 5.6 million people, 50% of whom older than 80 years.^2,3^

Several clinical situations, such as hypertension, heart failure, rheumatic and non-rheumatic valvular diseases, diabetes mellitus and hyperthyroidism, increase the risk for AF.^4^

The most common clinical manifestations of symptomatic patients with AF are palpitations, dizziness, dyspnea, chest pain and reduced tolerance to physical activity. Less specific symptoms, such as fatigue, anxiety and depression, have also been reported. Therefore, the physical or emotional clinical manifestations of patients with AF have a substantial impact on their quality of life.^5-9^

Although the impact on the quality of life of patients with AF is recognized, few health care services systematically assess that impact to follow those patients up.^10,11^

Assessing the quality of life by using specific scientifically validated instruments can contribute to objectively characterize the phenomenon.^12^ To introduce that type of approach in the medical practice and that of all other health care professionals, simplified questionnaires suitable for the major clinical manifestations, in addition to the patients' emotional responses to the disease process, should be developed. That assessment implies the contemplation of the major dimensions of life, comprising physical, social and emotional domains.^13^ From that perspective, the assessment with a specific instrument might even aid with decision making regarding therapy.^9^

In Brazil, a quality of life questionnaire for AF patients (AFQLQ version 1 - AFQLQ v.1) was developed and validated, aiming at assessing the major clinical manifestations and treatments in the Brazilian population with AF.^14^ After critical review, we proposed an update of that questionnaire by including the new domains of fatigue, illness perception and well-being.

This study analyzes the consistency and reproducibility of the AFQLQ v.1 after including those new domains (AFQLQ version 2 - AFQLQ v.2).

## Methods

This is a longitudinal study carried out in the Anticoagulation Outpatient Clinic for AF Patients, in the UNIFESP university-affiliated hospital.

This study sample consisted of 40 patients, older than 18 years, with persistent or permanent AF of any etiology, referred for anticoagulation control (Table 1).

**Table 1:** Description of patients according to the atrial fibrillation (AF) type, time since diagnosis, CHADS score, interventions performed and knowledge of the arrhythmia. São Paulo, 2013

	**n**	**%**
Type of AF		
Persistent	19	47.5
Permanent	21	52.5
Time since diagnosis		
up to 1 year	14	35
1 - 5 years	16	40
6 - 10 years	5	12.5
11 or more years	5	12.5
CHADS_2_		
0	0	0
1	30	75
2	9	22.5
3	1	2.5
EF*		
> 50%	22	55
< 50%	5	12.5
Left atrial size*		
Mean ± SD	41.4 ±1.2	
Electrical cardioversion		
Yes	21	52.5
No	19	47.5
Ablation		
Yes	4**	10
No	36	90
Knowledge of the arrhythmia		
Yes	15	37.5
No	25	62.5

EF: ejection fraction.

* Echocardiographic values not calculated for 13 patients (32.5%).

** Patients underwent electrical cardioversion and ablation

The following data were obtained from the patients' responses to the questionnaire and from the information originating from the medical records: time elapsed since the diagnosis of AF; CHADS_2_ and CHA_2_DS_2_-vasc scores; use of devices; echocardiographic data (ejection fraction and left atrial area); AF classification (permanent or persistent); medications used; and patients' knowledge of the name of the arrhythmia. Quality of life was measured by using the questionnaires SF-36 (*Medical Outcome Study Short-Form Health Survey*), AFQLQ v.1 and AFQLQ v.2. All information was collected at the first visit (Table 1).

The SF-36 is a generic questionnaire. Translated and validated in Brazil by Ciconelli et al. in 1999, it is composed of 36 questions, gathered in 8 domains that reflect social and physical and mental health aspects. Each domain's score ranges from 0 to 100 points, and the higher the score, the better the quality of life.^15^

The AFQLQ v.1 was developed by Bragança et al.^14^ in 2010, and comprised 22 questions and 82 items in 7 domains. It was aimed at assessing the major clinical manifestations and treatments in a simplified way. The total score varies from 0 to 100 points, and the lower the score, the better the quality of life.^14^

The critical analysis of the use of the AFQLQ in the UNIFESP Outpatient Clinic of Arrhythmias identified the need for the implementation of new domains, as well as the withdrawal of the domain 'therapy', which had always been the reason for critics and controversies in assessing the result.

Therefore, in the AFQLQ v.2, the 7 domains were maintained, the questions passing from 22 to 30, and the items, from 82 to 134. The total score varies from 0 to 140 points, and the lower the score, the better the quality of life. The transition questions with 'yes' or 'no' response were neither enumerated nor scored. All domains have the same score value, 20 points, were sequentially enumerated from I to VII, in Roman numerals, and comprised the major clinical AF manifestations described in the AFQLQ v.1 (palpitation, dyspnea, chest pain and dizziness) added by fatigue, well-being and illness perception.

To estimate reproducibility or reliability, test-retest and internal consistency methods were used as follows:

-Intraobserver reproducibility: Observer 1 applied 21 AFQLQ v.2 and SF-36 to patients followed up in the Anticoagulation Outpatient Clinic for AF Patients. After 15 days, the same observer reapplied those questionnaires to the same patients.
-Interobserver reproducibility: Observer 1 applied 19 AFQLQ v.2 and SF-36 to patients followed up in the Anticoagulation Outpatient Clinic for AF Patients. After 15 days, observer 2 reapplied those questionnaires to those same patients.

This study was approved by the UNIFESP Ethics Committee (n.0852/10), and all participants provided written informed consent according to the 466/12 National Board of Health Resolution.

### Statistical analysis

The scores of the SF-36 and AFQLQ v.2 domains, the AFQLQ v.2 total score and the clinical variables were grouped in a data bank of the SPSS (Statistical Packcage Social Science) software, version 19. Initially the descriptive statistics of the patients' demographic and clinical conditions was performed, and the distribution of the absolute and relative frequencies of the categorical variables was obtained. The continuous variables were presented as mean ± standard deviation.

**Test-retest.** The stability of the attribute 'quality of life' in the 15-day interval was tested by comparing the means obtained in the initial assessment and in the second assessment by using paired *t* test and Spearman correlation. To assess the reliability of the new version of the AFQLQ, Bartko intraclass correlation coefficient (ICC) was used in the total sample of patients between the different observers. A measuring instrument is considered to have good reliability for ICC values higher than 0.80. The paired *t* test assesses the mean scores obtained in two applications. A statistically significant difference indicates low reliability of the instrument.

**Internal consistency.** Internal consistency was determined by using Cronbach's alpha coefficient. The following were estimated: item-total correlation; total alpha value; and alpha value of each item. Scores greater than 0.8 are desirable, and greater than 0.90, excellent.^16^

To reject the null hypothesis, the significance level of p < 0.05 (5%) was adopted. The 95% confidence intervals were calculated.

Similarly to the previous version, we assumed that the quality of life of the patients studied has a parametric distribution, thus the normality test was not used.^14^

## Results

Of the patients assessed, 26 (65%) were of the male sex, and the patients' age varied from 43 to 86 years (mean of 61.2 ± 9.6 years).

Regarding the characteristics of AF, 47.5% of the patients had persistent AF, and 52.5%, permanent AF. Regarding the time elapsed since diagnosis, 35% had the disease for up to one year, 40% had it from one to five years, and 25%, for more than five years. Regarding risk classification, most patients were CHADS1 and 2 (67.5% and 22.5%, respectively). The mean left atrial size was 41.4 ± 1.2 mm. Regarding therapy, 52.5% had already undergone electrical cardioversion, and 10% had undergone catheter ablation. When asked about knowledge of the disease, 25% reported none (Table 1).

The new version of the AFQLQ maintained the same structure and metric of the original (appendix). Similar to the original, the AFQLQ v.2 remained easy to apply and to understand. This information originated from the assessment of the focal group during the development of version 2.

The AFQLQ v.2 score system remained objective, simple and fast. An individualized score for each domain was maintained, as was a total/global value for the addition of the values found in each domain.

Table 2 shows the reproducibility assessment of AFQLQ v.2, correlating the scores of the initial application with those of the test-retest after 15 days. Inter- and intraobserver reproducibilities can be demonstrated by using accurate coefficients (≥ 0.90) in the analysis of the total score of AFQLQ v.2. The Bartko ICC was greater than 0.95 for the total score of AFQLQ v.2, implying high accuracy. The ICC of the domains 'fatigue', 'well-being' and 'illness perception' had values greater than 0.85 for the reproducibility analysis.

**Table 2:** Reproducibility of AFQLQ v.2 according to intraclass correlation coefficient (ICC) between the scores of the initial test and of the retest after 15 days. São Paulo, 2013

		**ICC (Bartko)**	
	**Test-retest ( n=40)**	**Intraobserver ( n=21)**	**Interobserver (n=19)**
Total score	0.98	0.98	0.97
Palpitation	0.84	0.94	0.71
Dyspnea	0.82	0.95	0.75
Chest pain	0.81	1.00	0.72
Dizziness	0.56	0.69	0.48
Fatigue	0.92	0.89	0.94
Well-being	0.87	0.90	0.85
Illness perception	0.88	0.87	0.89

Similarly to the original version, which showed excellent internal consistency for reproducibility,^14^ the internal consistency of the new version also showed highly reliable results of agreement: Cronbach's alpha > 0.82. The item-by-item assessment showed correlation greater than 0.75 between the total result of the test and the domain 'well-being' (Cronbach's alpha = 0.76). Similar results were found for the domains 'fatigue' and 'illness perception', with Cronbach's alphas of 0.78 and 0.79, respectively (Table 3).

**Table 3:** Item-total correlation coefficient, total alpha value of the 7 domains of AFQLQ v.2, and alpha values of each item. São Paulo, 2013

	**Item-total correlation coefficient**	**Cronbach's alpha, if item excluded**
AFQLQ v.2 - Total		0.82
Palpitation	0.54	0.80
Dyspnea	0.47	0.81
Chest pain	0.47	0.81
Dizziness	0.44	0.82
Fatigue	0.68	0.78
Well-being	0.76	0.76
Illness perception	0.61	0.79

## Discussion

The construction of an instrument to assess quality of life and its validation require several steps, reproducibility or reliability being one of the most important ones in psychometric assessment, fundamental to the construction of that type of instrument.^17^ For a questionnaire to be valid, it does not suffice to measure what it is intended to. It has to reproduce the same findings when used in other scenarios and by other observers. That result is determined by the reproducibility or reliability analysis.^17,18^

Quality of life is a multifactorial construct, thus, measuring it by use of generic instruments reduces its assessment power. The generic instruments for assessing quality of life establish several health conditions and reflect different areas of people's life. However, it falls short when the intention is to measure the real impact on the quality of life, because the particularities of certain clinical situations cannot always be assessed or are underestimated.^11^

The development of specific questionnaires for several disease manifestations, thus, has been widespread, especially in the cardiovascular area.^19-23^

One question is pertinent: Does the use of international instruments adapted to our language empower us, or should we use validated national instruments that contemplate our population's characteristics? What is easier and real?

AF is the most frequent cardiac arrhythmia in Brazil. It has an adverse prognosis, because it increases the risk for stroke, and, consequently, mortality. AF is not restricted to an electrocardiographic change; it also involves other physical and psychic manifestations, which affect the quality of life, in some or all of its dimensions. Therefore, the need to measure the impact of AF on quality of life is unquestionable.^24^

Measuring all aspects involved with the quality of life of individuals with AF remains a great challenge, because the symptoms of that arrhythmia can prevent patients from maintaining their daily and/or social activities, contributing to the deterioration of their emotional health. This explains the existence of several questionnaires for AF in other idioms, but they measure mainly the clinical manifestations, jeopardizing the global assessment of quality of life.^10,11,18,25^

With that in mind, improving the original version of the questionnaire (AFQLQ v.1) by adding new domains, which are less common to the clinical practice, allows the questionnaire to meet the objective of quality of life assessment in its many dimensions in the Brazilian population.

The new version of AFQLQ perfected the previous one, confirming its specificity for patients with AF and more reliably reflecting their reality. Excluding the domain 'therapy', which contemplated interventions with drugs, cardioversion and ablation, and including manifestations, such as fatigue, illness perception and well-being, allowed the questionnaire to assess individuals without the potentially confounding factors in assessing the impact of AF *per se*. This does not exclude the possibility of using the questionnaire to analyze the treatments for AF and their responses concerning quality of life.

The inclusion of the domain 'fatigue', although a subjective symptom of no metric confirmation, is crucial, because it appears as the third most frequently found symptom in patients with AF, significantly influencing their functional capacity and physical activity limitation. However, that domain is sometimes assessed as a manifestation related to intolerance to exercise/exertion, which is not true.^9,25-28^

Similarly, including subjective components, such as well-being and illness perception, in addition to enabling the assessment with clear questions, provided support to assess the individual in other dimensions.^29-34^

A recent systematic review has shown the concern of health care professionals with the relationship between the emotional and physical aspects of patients with AF. That review, comprising 34 studies specifically assessing anxiety and depression, has concluded that there is a complex relationship between the symptoms of depression and anxiety and the clinical manifestations of AF.^30^

Regarding illness perception, patients and physicians differ about the threat AF represents to health, as well as morbidity and mortality. Physicians tend both to underestimate the understanding of patients about the benefit derived from treatment, and to overestimate their knowledge of the complications.^35^

In accordance with the literature, the new version provides quality of life assessment, domain-by-domain, both separately and globally: Are there symptoms? If the response is positive, which are they? Can well-being or illness perception influence quality of life? Which set of domains has the greater influence?^36^

A specific and simplified questionnaire, such as AFQLQ v.2, enables assessing whether physical symptoms affect well-being and/or emotional health and vice-versa. Not assessing all the quality of life dimensions of individuals with AF can lead us to the reductionist management of considering only heart rate or rhythm control in those patients. We believe that hinders a more holistic approach to the disease.

The possibility of incorporating the questionnaire into the routine visit to any health care professional, because of its rapid application, makes it a practical tool in the assessment of patients with AF.

It is worth noting that the use of a simple and direct language allows the AFQLQ v.2 to be applied to patients of any educational level. The easily applicable score system provides results on the quality of life rapidly and of easy interpretation.

The strict analysis of the psychometric properties of this new version of the AFQLQ, with statistically significant reproducibility as compared to that of the gold-standard, the SF-36, ensures the suitability of the AFQLQ v.2 to be used in assessing the quality of life of patients with AF.

## Conclusion

The new version of the AFQLQ proposed in the present study (AFQLQ v.2) showed not only improvement of the original version, but maintained high reproducibility. These characteristics are essential to the clinical applicability of that instrument.

### Study limitations

The validation of this new version of the AFQLQ did not classify the participants into their socioeconomic levels. This new version of the AFQLQ requires further follow-up studies in the outpatient clinic to confirm its sensitivity to detect oscillations in the patients' quality of life paralleling their clinical improvement or worsening.

## Appendix Avaliação da Qualidade de Vida em Pacientes com Fibrilação Atrial versão 2

**Table:**

Identification		Date:	N:
Name:			
ID:	Sex:	Birth date:	Age
Natural:	Proced:	Profession:	
Marital status:		Lives with:	
Nível de escolaridade:	( ) Illiterate	( ) Incomplete elementary	( ) Complete elementary
	( ) Incomplete middle	( ) Complete middle	( ) Incomplete college
	( ) Complete college		
Knowledge of the name of the arrhythmia? ( ) yes ( ) no			

### Questionnaire

**1. Palpitation**

1. Do you have palpitation?
  ( ) Yes ( ) No
  Palpitation occurs:
    ✓Daily .............................................................................................................e
  ✓Weekly ..........................................................................................................d
  ✓Fortnightly ....................................................................................................c
  ✓Monthly..........................................................................................................b
  ✓ >30 days........................................................................................................a
2. Palpitation appears:
  ✓ At rest...........................................................................................................c
  ✓ With emotional stress...................................................................................b
  ✓ With common activities (at work or at home)..............................................a
3. How long does palpitation last?
  ✓ < 1 minute.....................................................................................................a
  ✓ Minutes.........................................................................................................b
  ✓ Hours............................................................................................................d
  ✓ Continuously (direct)....................................................................................e
4. When has the last palpitation begun?
  ✓ < 48 hours.....................................................................................................c
  ✓ > 48 hours - 1 week......................................................................................k
  ✓ > 1 week - 1 month.......................................................................................b
  ✓ > 1 month - 6 months....................................................................................l
  ✓ > 6 months - 1 year.......................................................................................a
  ✓ > 1 year.........................................................................................................m
5. How does palpitation hinder your daily routine?
  ✓ Extremely......................................................................................................d
  ✓ Somewhat.....................................................................................................c
  ✓ Slightly..........................................................................................................b
  ✓ Nothing at.....................................................................................................a
  **II. Dyspnea**
  Do you have *shortness or breathlessness*?
  ( ) Yes ( ) No
6. *Shortness of breath* occurs:
  ✓ At rest .....................................................................................................................f
  ✓ On effort:
  - minimum .......................................................................................................e
  - medium ..........................................................................................................d
  - large ...............................................................................................................b
7. It is accompanied by:
  ✓ Palpitation ....................................................................................................b
  ✓ Cough ...........................................................................................................a
  ✓ Dizziness ......................................................................................................b
  ✓ Others ...........................................................................................................a
  ✓ No symptom .................................................................................................z
8. How does *shortness of breath* hinder your daily routine?
  ✓ Extremely ...................................................................................................h
  ✓ Somewhat ....................................................................................................f
  ✓ Slightly .......................................................................................................d
  ✓ Nothing at all ...............................................................................................a
    **III. Chest pain**
    Do you have chest pain?
    ( ) Yes ( ) No
9. Has it begun at the time of palpitation?
  ( ) Yes ( ) No
10. Chest pain appears:
  ✓ At rest ..........................................................................................................d
  ✓ With emotional stress ..................................................................................c
  ✓ With common activities (at work or at home) .............................................b
  ✓ Other ............................................................................................................a
11. Chest pain is accompanied by:
  ✓ No symptom .................................................................................................z
  ✓ Sweating ......................................................................................................b
  ✓ Nausea and/or vomiting ...............................................................................b
  ✓ Radiation ......................................................................................................a
  ✓ Other ............................................................................................................a
12. How does chest pain hinder your daily routine?
  ✓ Extremely ....................................................................................................g
  ✓ Somewhat ....................................................................................................e
  ✓ Slightly ........................................................................................................c
  ✓ Nothing at all ...............................................................................................a
  **IV. Dizziness**
    Do you have dizziness?
    ( ) Yes ( ) No
13. How is your dizziness?
  ✓ Transient sensation of unbalance .................................................................c
  ✓ Rotational movement: you or the environment ...........................................c
  ✓ Fainting sensation ........................................................................................d
  ✓ Fainting ........................................................................................................f
14. Dizziness is accompanied by:
  ✓ Palpitation ....................................................................................................b
  ✓ Blurred vision ..............................................................................................b
  ✓ Anxiety ........................................................................................................a
  ✓ Weakness .....................................................................................................a
  ✓ Others ..........................................................................................................a
  ✓ No symptom ................................................................................................z
15. How does dizziness hinder your daily routine?
  ✓ Extremely ....................................................................................................g
  ✓ Somewhat ....................................................................................................e
  ✓ Slightly ........................................................................................................c
  ✓ Nothing at all ...............................................................................................a
  **V. Fatigue**
    Do you have fatigue (tiredness, weakness, lack of energy)?
    ( ) Yes ( ) No
  Do you believe your fatigue relates to your arrhythmia?
    ( ) Yes ( ) No
16. Fatigue occurs:
  ✓ Daily ............................................................................................................d
  ✓ Weekly .........................................................................................................c
  ✓ Continuously ................................................................................................e
  ✓ Sporadically .................................................................................................a
17. You consider your fatigue:
  ✓ Mild .............................................................................................................a
  ✓ Moderate .....................................................................................................b
  ✓ Severe ..........................................................................................................c
18. Fatigue is accompanied by:
  ✓ No symptom ................................................................................................z
  ✓ Shortness of breath .....................................................................................b
  ✓ Palpitation ...................................................................................................b
  ✓ Others ..........................................................................................................a
  ✓ More than one symptom .............................................................................e
19. Your fatigue relates to:
  ✓ Nothing ........................................................................................................e
  ✓ Daily activity ...............................................................................................c
  ✓ Emotional status ..........................................................................................b
20. How does fatigue hinder your daily routine?
  ✓ Extremely ....................................................................................................d
  ✓ Somewhat ....................................................................................................c
  ✓ Slightly ........................................................................................................b
  ✓ Nothing at all ...............................................................................................z
  **VI. Well-being**
    You feel that the arrhythmia CURRENTLY affects:
21. Your joy:
  ✓ Extremely ....................................................................................................d
  ✓ Somewhat ....................................................................................................c
  ✓ Slightly ........................................................................................................a
  ✓ Nothing at all ...............................................................................................z
22. Your sadness:
  ✓ Extremely ....................................................................................................d
  ✓ Somewhat ....................................................................................................c
  ✓ Slightly ........................................................................................................a
  ✓ Nothing at all ...............................................................................................z
23. Your anxiety, irritation, nervousness:
  ✓ Extremely ....................................................................................................d
  ✓ Somewhat ....................................................................................................c
  ✓ Slightly ........................................................................................................a
  ✓ Nothing at all ...............................................................................................z
24. Your optimism:
  ✓ Extremely ....................................................................................................d
  ✓ Somewhat ....................................................................................................c
  ✓ Slightly ........................................................................................................a
  ✓ Nothing at all ...............................................................................................z
25. Your pessimism:
  ✓ Extremely ....................................................................................................d
  ✓ Somewhat ...................................................................................................c
  ✓ Slightly ........................................................................................................a
  ✓ Nothing at all ...............................................................................................z
  **VII. Illness perception - arrhythmia:**
    Do you believe you will be cured?
    ( ) Yes ( ) No
26. How long do you think your arrhythmia will last?
  ✓ Short time ....................................................................................................a
  ✓ Long time ....................................................................................................b
  ✓ Forever ........................................................................................................c
27. How does taking the medication hinder your daily routine?
  ✓ Extremely ....................................................................................................e
  ✓ Somewhat ...................................................................................................d
  ✓ Slightly ........................................................................................................c
  ✓ Nothing at all ...............................................................................................z
28. How much concerned are you with your arrhythmia?
  ✓ Extremely ....................................................................................................d
  ✓ Somewhat ....................................................................................................c
  ✓ Slightly ........................................................................................................a
  ✓ Nothing at all ...............................................................................................z
29. To what extent does your arrhythmia affect you emotionally?
  ✓ Extremely ....................................................................................................d
  ✓ Somewhat ....................................................................................................c
  ✓ Slightly ........................................................................................................a
  ✓ Nothing at all ...............................................................................................z
30. Do you relate this arrhythmia to your death/end of life?
  ✓ Several times ...............................................................................................d
  ✓ Sometimes ...................................................................................................c
  ✓ Rarely ..........................................................................................................a
  ✓ Never ...........................................................................................................z

**Table:**

**A**	**1.0**	**H**	**8.0**
**B**	**2.0**	**I**	**9.0**
**C**	**3.0**	**J**	**10.0**
**D**	**4.0**	**K**	**2.5**
**E**	**5.0**	**L**	**1.5**
**F**	**6.0**	**M**	**0.5**
**G**	**7.0**	**Z**	**0.0**
